# A Case of Noncompaction at All Segments of Both Right and Left Ventricles

**DOI:** 10.1155/2014/325257

**Published:** 2014-11-26

**Authors:** Ali Pourmoghaddas, Reihaneh Zavar, Mohaddeseh Behjati

**Affiliations:** ^1^Cardiology, Isfahan University of Medical Sciences, Isfahan, Iran; ^2^Heart Failure Research Center, Cardiovascular Research Center, Cardiovascular Research Institute, Isfahan University of Medical Sciences, Isfahan, Iran

## Abstract

*Background*. Noncompaction/hypertrabeculation left ventricle (NCM/HVM) is most commonly reported in one or more segments of left ventricle and sometimes both ventricles. In this case, we present noncompaction of all segments of right and left ventricle, in a young man with mental retardation. *Case Presentation*. A 19-year-old male was referred to us with sudden dyspnea at rest and chest discomfort. He was a known case of mental retardation. He was born full term with birth weight = 1250 grams. On physical examination. A systolic murmur (II/VI) at left sternal border was heard. ECG showed increased voltage in precordial lead and deep ST segment depression. Chest X-ray (CXR) was within normal limits. Transthoracic echocardiography showed situs solitus, D loop, normal connection of great vessels, noncompaction LV at all segments (noncompaction/compaction = 2.5/0.5) with moderate systolic dysfunction (LVEF = 40%), diastolic dysfunction grade II, normal RV size with mild systolic dysfunction and hypertrabeculation, mild tricuspid regurgitation (TR), and normal pulmonary artery systolic pressure. After injection of agitated saline some bubbles were passed from right to left through patent foramen oval (PFO). *Conclusions*. Extensive sinusoid formation and trabeculation of RV and nearby all LV segments and its association with mental retardation suggest presence of strong genetic background.

## 1. Introduction

Noncompaction/hypertrabeculation left ventricle (NCM/HVM) occurs consequent to disorder of endomyocardial morphogenesis due to the failure of trabecular compaction during development of myocardial tissue [1]. This congenital abnormality is characterized by numerous sinusoidal or excessive trabeculae and deep intratrabecular recesses covered by endothelial cells which continued with ventricular endothelium [2]. Precise recognition of this disorder is highly warranted due to its high rate of morbidity and mortality attributed to progressive heart failure, malignant arrhythmia, and thromboembolism [3]. By now, infliction of one or more segments of left ventricle and sometimes both ventricles has been described [4]. Indeed, there are no available criteria for noncompaction in the right ventricle. Hereby, we present a case of a combination of right and left ventricle NCM/HVM in all segments in a young man with mental retardation.

## 2. Case Presentation

**Figure 1:**
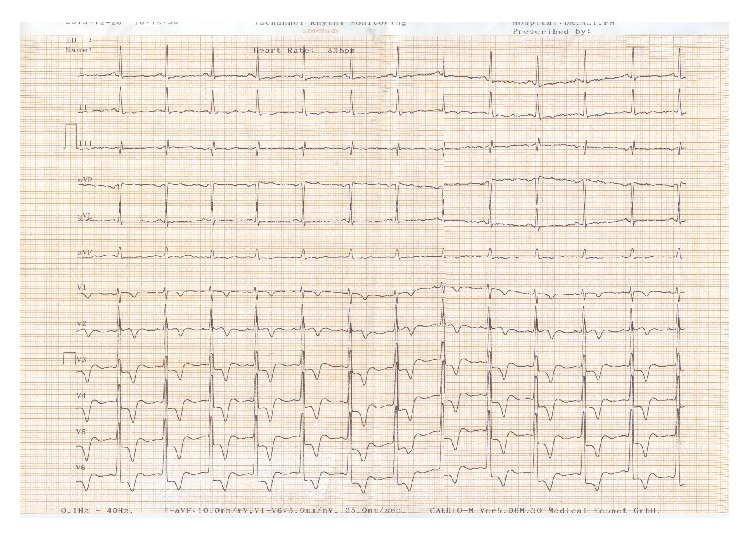
ECG high voltage QRS, ST depression, and T inversion.

**Figure 2:**
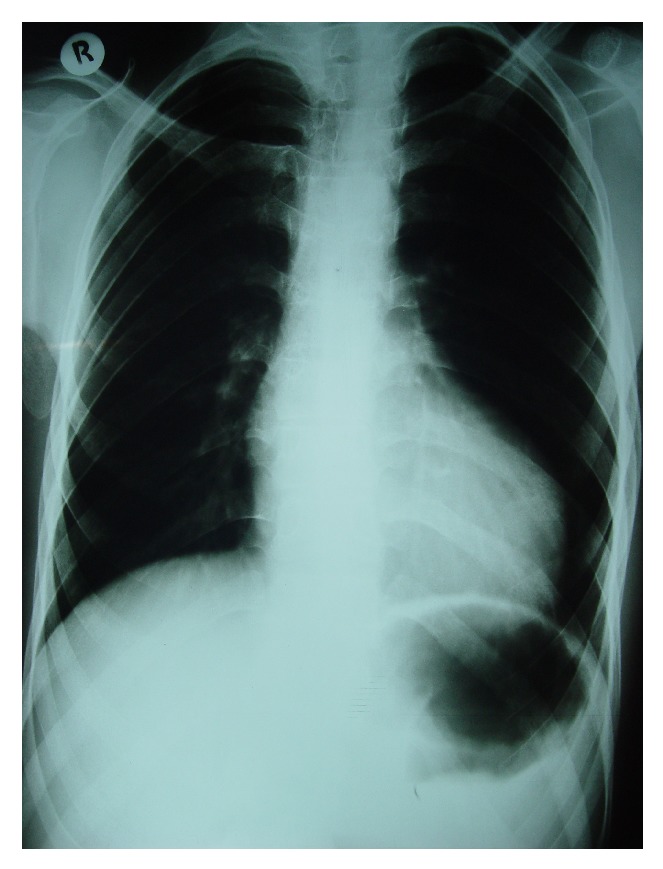
CXR CTR is normal.

**Figure 3:**
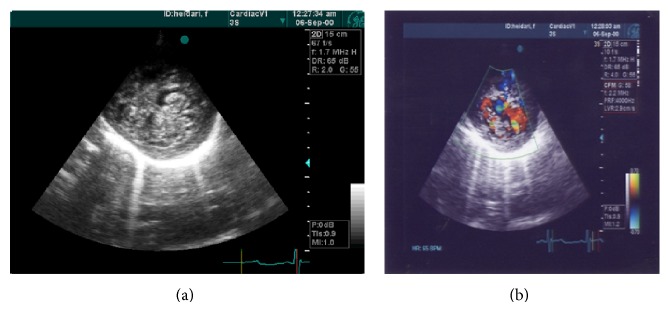
SAX view (thickness noncompaction/compaction = 2.5/0.5).

**Figure 4:**
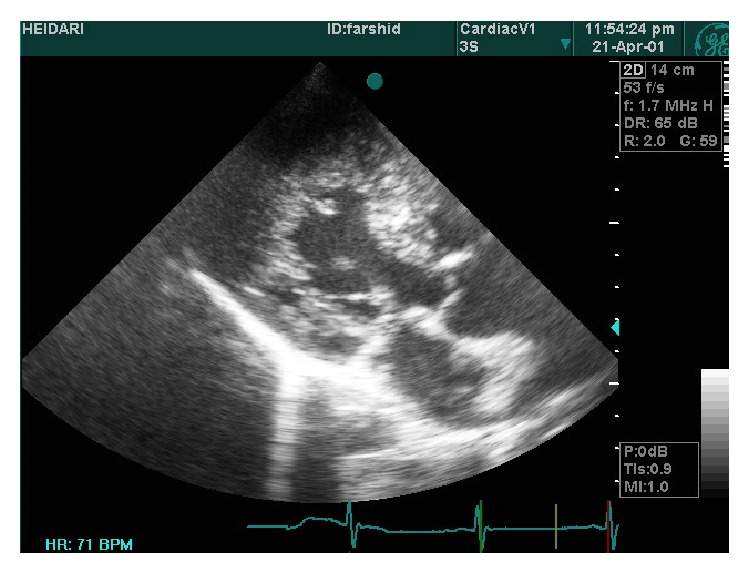
Parasternal long axis view showed hypertrabeculation, antroseptal and posterior wall.

**Figure 5:**
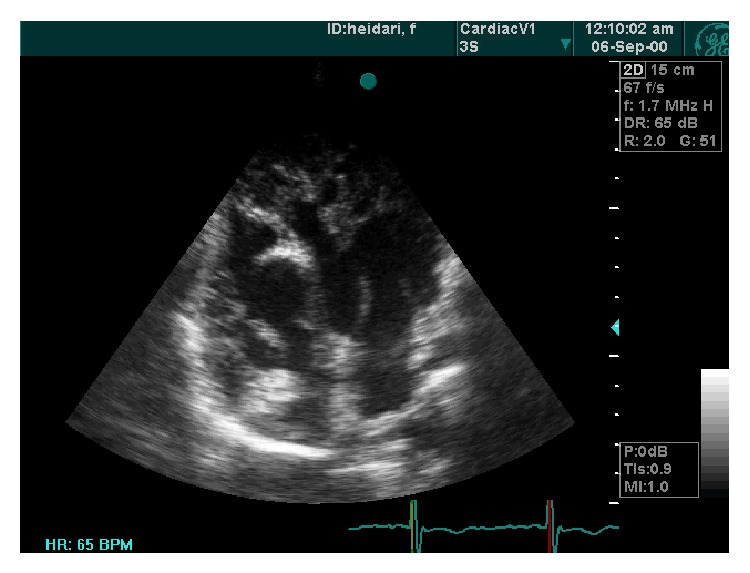
4C view of prominent hypertrabeculation in apical segments and less in lateral wall.

**Figure 6:**
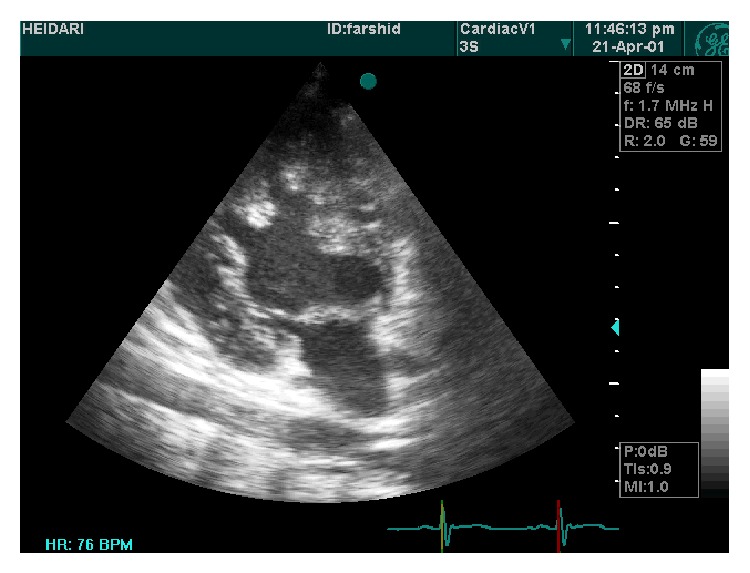
2C view of hypertrabeculation in inferior wall and anterior.

**Figure 7:**
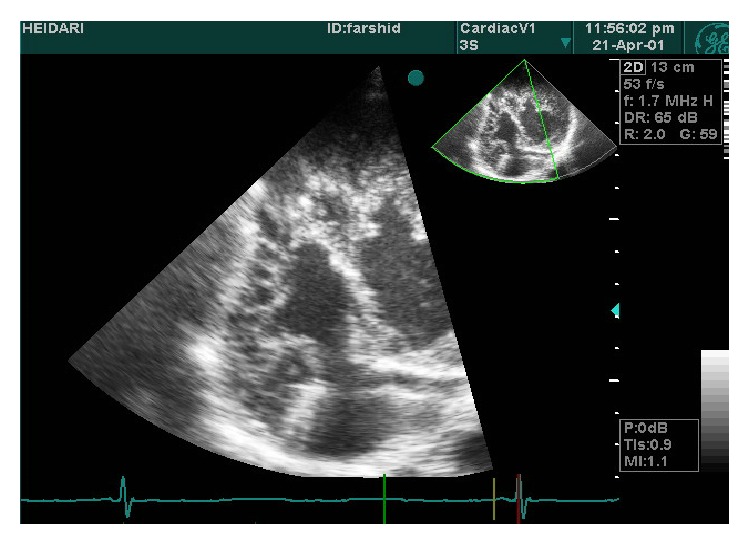
Hypertrabeculation in RV.

A 19-year-old male was referred to ED of Chamran Hospital, Isfahan University of Medical Sciences, Isfahan, Iran, due to sudden dyspnea at rest and chest discomfort. He was a known case of mental retardation. He was born full term with birth weight = 1250 grams. On physical examination, silent pericardium and percussion dullness from second to fifth left intercostal space were detected. A systolic murmur (II/VI) at left sternal border was heard. ECG showed giant voltages in precordial lead and deep ST segment depression (Figure 1). Chest X-ray (CXR) was within normal limits (Figure 2). Transthoracic echocardiography (Figures 3, 4, 5, 6 and 7) showed situs solitus, D loop, normal connection of great vessels, noncompaction LV at all segments (noncompaction/compaction = 2.5/0.5) with moderate systolic dysfunction (LVEF = 40%), diastolic dysfunction grade II, normal RV size with mild systolic dysfunction and hypertrabeculation, mild tricuspid regurgitation (TR), and normal pulmonary artery systolic pressure. After injection of agitated saline some bubbles were passed from right to left through patent foramen ovale (PFO). He was scheduled for close observation.

## 3. Discussion

NCM/HVM is a rare congenital cardiomyopathy in which apical-lateral and basal-septal segments are most and least commonly affected walls, respectively [4]. Interruption of normal myocardial compaction from basal-septal to the apical-lateral segments during embryogenic development has been suggested for this pattern of wall involvement [5]. Noncompaction myocardium differs from “persistent sinusoids” seen with other congenital heart diseases as pulmonary atresia, in which cardiac chambers communicate with epicardial circulation [1]. Heart failure is the most common presentation of NCM which manifests by progressive dyspnea on exertion, orthopnea, and lower extremity edema [6]. By now, at least seven types of spongy or fetal myocardium are defined as isolated noncompaction, isolated noncompaction with arrhythmias, dilated form of noncompaction, hypertrophic form, restrictive form, hypertrophic and dilated form, and noncompaction with congenital heart disease as atrial septal defect, ventricular septal defect, pulmonary stenosis, pulmonary artery abnormality, right and left ventricular outflow tract obstruction, and left heart hypoplastic syndrome. Association with dysmorphic syndrome has been reported in 10% of cases [1]. Disease reported in pediatric population has poorer outcome compared with adult population [7]. Overall prognosis is highly related to the ejection fraction which is inversely related to the number of noncompacted segments [8].

Our case showed typical echocardiographic patterns of NCM as two-layered myocardial tissue with a thin compacted outer layer, a thinner noncompacted inner band, and deep myocardial trabeculation. By color Doppler imaging, at least three deep intratrabecular recesses which communicate with ventricular cavity should be identified. A noncompaction/compaction ratio >2.0 measured in systole is considered a diagnostic measure [5]. Trabecular regions associated with NCM are segmental rather than diffuse as in the case of left ventricular hypertrophy. Interestingly, this case shows NCM in both ventricles and all segments show trabeculation tissue equally. This case describes the clinical scenario of NCM of RV and nearby all LV segments and its association with mental retardation. Moderate LV systolic dysfunction in this case could be related to the important base of cardiomyopathy and renders suitable therapies. To the best of our knowledge, this is the first presentation of association between extensive NCM and mental retardation.

## 4. Conclusions

Extensive sinusoid formation and trabeculation of RV and nearby all LV segments and its association with mental retardation suggest presence of strong genetic background.

## Supplementary Material

Non compaction/hyper trabeculation left ventricle (NCM/HVM) is most commonly reported in one or more segments of left ventricle and sometimes both ventricles. In this case, we present non compaction of all segments of right and left ventricle, in a young man with mental retardation.
